# Both Strands of siRNA Have Potential to Guide Posttranscriptional Gene Silencing in Mammalian Cells

**DOI:** 10.1371/journal.pone.0005382

**Published:** 2009-04-29

**Authors:** Jun-Xia Wei, Jie Yang, Ji-Feng Sun, Lin-Tao Jia, Yong Zhang, Hui-Zhong Zhang, Xia Li, Yan-Ling Meng, Li-Bo Yao, An-Gang Yang

**Affiliations:** 1 State Key Laboratory of Cancer Biology; Department of Biochemistry and Molecular Biology, Fourth Military Medical University, Xi'an, China; 2 Department of Clinical Diagnosis, Tangdu Hospital, Fourth Military Medical University, Xi'an, China; 3 Department of Nephrology, Tangdu Hospital, Fourth Military Medical University, Xi'an, China; 4 Department of Immunology, Fourth Military Medical University, Xi'an, China; University of Helsinki, Finland

## Abstract

Despite the widespread application of RNA interference (RNAi) as a research tool for diverse purposes, the key step of strand selection of siRNAs during the formation of RNA-induced silencing complex (RISC) remains poorly understood. Here, using siRNAs targeted to the complementary region of Survivin and the effector protease receptor 1 (EPR-1), we show that both strands of the siRNA duplex can find their target mRNA and are equally eligible for assembly into Argonaute 2 (Ago2) of RISC in HEK293 cells. Transfection of the synthetic siRNA duplexes with different thermodynamic profiles or short hairpin RNA (shRNA) vectors that generate double-stranded RNAs (dsRNAs), permitting processing specifically from either the 5′ or 3′ end of the incipient siRNA, results in the degradation of the respective target mRNAs of either strand of the siRNA duplex with comparable efficiencies. Thus, while most RNAi reactions may follow the thermodynamic asymmetry rule in strand selection, our study suggests an exceptional mode for certain siRNAs in which both strands of the duplex are competent in sponsoring RNAi, and implies additional factors that might dictate the RNAi targets.

## Introduction

RNA interference (RNAi) is a gene-silencing process during which endogenous messenger RNA (mRNA) is destroyed by introduced corresponding double-stranded RNA [1]. RNAi has found widespread application as a technique in research laboratories, since it permits the simple yet effective knockdown of genes of interest. RNAi-related processes are physiologically critical for development and heterochromatin formation, and offer cellular protection against virus and transposon amplification [2]. Despite the widespread use of RNAi for the knockdown of genes, the RNAi pathway, especially the detailed mechanisms underlying the formation of RNA-induced silencing complex (RISC), remains poorly understood.

Small interfering RNAs (siRNAs) were first identified as the specificity determinants of the RNAi pathway, wherein they act as guides that direct the endonucleolytic cleavage of their target RNAs. Prototypical siRNA duplexes are 21 nucleotide (nt) double-stranded RNAs (dsRNAs), containing 19 base pairs and 2-nt 3′ overhangs [3]–[5].

The results of several in vitro experiments indicate that only one strand of the siRNA duplex is loaded onto RISC, which in turn uses this strand as the guide RNA to find complementary mRNA sequences via Watson-Crick base pairing and cleaves the phosphodiester bond between the 10th and 11th nucleotides in the target molecules via an endonucleolytic pathway as measured from the 5′ end of the guide strand. Although it is reported that the selection of the guide strand is based on the rule of thermodynamic asymmetry, the way selected guide strand is released from the double-stranded siRNA and the fate of the anti-guide strand remains unclear [2], [6], [7], [8], [9]. It also remains to be investigated whether the results obtained using in vitro RNAi reaction systems reflect the actual events occurring in mammalian cells. To illustrate the molecular mechanism of siRNA loading onto RISC and its subsequent activation process in cultured mammalian cells, we conducted a detailed biochemical analysis of this process. Our results are surprising, and as reported here, suggest an alternative model for siRNA loading.

Previous studies indicate that Argonaute 2 (Ago2), the essential mammalian member of the Argonaute protein family required for RISC assembly, recognizes the siRNA duplex rather than either of the single stands. The guide strand then directs the cleavage of the anti-guide strand via a process similar to the guide strand-directed cleavage of a target mRNA. The cleaved anti-guide strand is then dissociated and released [2], [6]. In a slicing RISC, the manner in which the cleavage products are unwound from the guide strand is unclear. Our data suggest that in mammalian cells, both strands of the siRNA duplex can direct RNAi. We thus propose that unwound siRNA duplexes yield two types of RISCs: one containing the antisense strand and the other containing the sense strand of the siRNA duplexes.

Survivin is a member of inhibitor of apoptosis (IAP) family, a gene family that plays important roles in apoptosis regulation. The Survivin gene is localized on chromosome 17 and contains 4 exons and 3 introns. Survivin is an onco-fetal protein, and is expressed in embryos and various malignant tumors. Effector protease receptor 1 (EPR-1), a protein that interacts with factor Xa in the vascular endothelium, is characterized by a long sequence in its mRNA that is complementary to the Survivin mRNA. The EPR-1gene is localized on chromosome 7 and encodes a protein with 337 amino acids [10]–[14]. The complementary characteristics of EPR-1 and Survivin provide a natural model for investigating the functions of the siRNA duplex and the formation of RISC in cultured mammalian cells.

In this study, by using cellular siRNA systems that targeted the complementary region of EPR-1 and Survivin mRNAs, we investigated the possibility of strand preference during siRNA incorporation into RISC and the subsequent binding of the target RNA. Similar to the data from an in vitro study on Drosophila which showed that siRNA strand selection was independent of dsRNA processing polarity during RNAi [15], our results revealed that in mammalian cells, RISC assembly and siRNA strand selection were not significantly influenced by the dsRNA processing step or thermodynamic profiles.

## Results

### EPR-1 and Survivin are coexpressed in the HEK293 cell line

**Figure 1:**
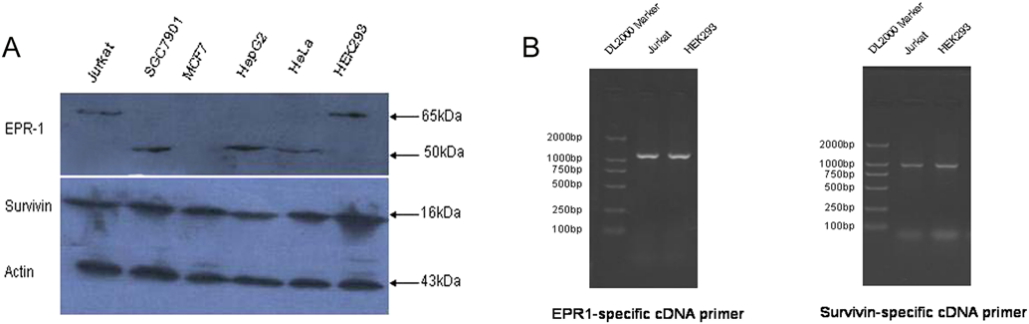
Screening for cell lines coexpressing both EPR-1 and Survivin. (A) The expressions of EPR-1 and Survivin in cell lines were analyzed by Western blotting using β-actin for standardization. (B) The expressions of EPR-1 and Survivin in HEK293 cell line were confirmed by RT-PCR using EPR-1 and Survivin specific cDNA primers.

As an initial test of our hypothesis, we examined the expression of EPR-1 and Survivin in several cell lines by using Western blotting with the aim of identifying a cell line that simultaneously expressed EPR-1 and Survivin. Our result showed that EPR-1 and Survivin were coexpressed in HEK293, SGC7901, HepG2, and HeLa cells (Figure 1). The molecular weight (MW) of EPR-1 in HEK293 cells is approximately 65 kDa, while in HepG2, SGC7901 and HeLa cells, it is ∼50 kDa. Reverse transcription-polymerase chain reaction (RT-PCR) using EPR-1-specific primers did not show any difference between these cell lines in terms of EPR-1 expression (data not shown). The difference in the MW of the protein is probably because the protein undergoes diverse posttranslational modifications. In our study, the HEK293 cell line was used for further investigations due to their relatively higher transfection efficacy.

### siRNAs targeted to the complementary region of EPR-1 and Survivin cause the simultaneous knockdown of both genes

**Figure 2:**
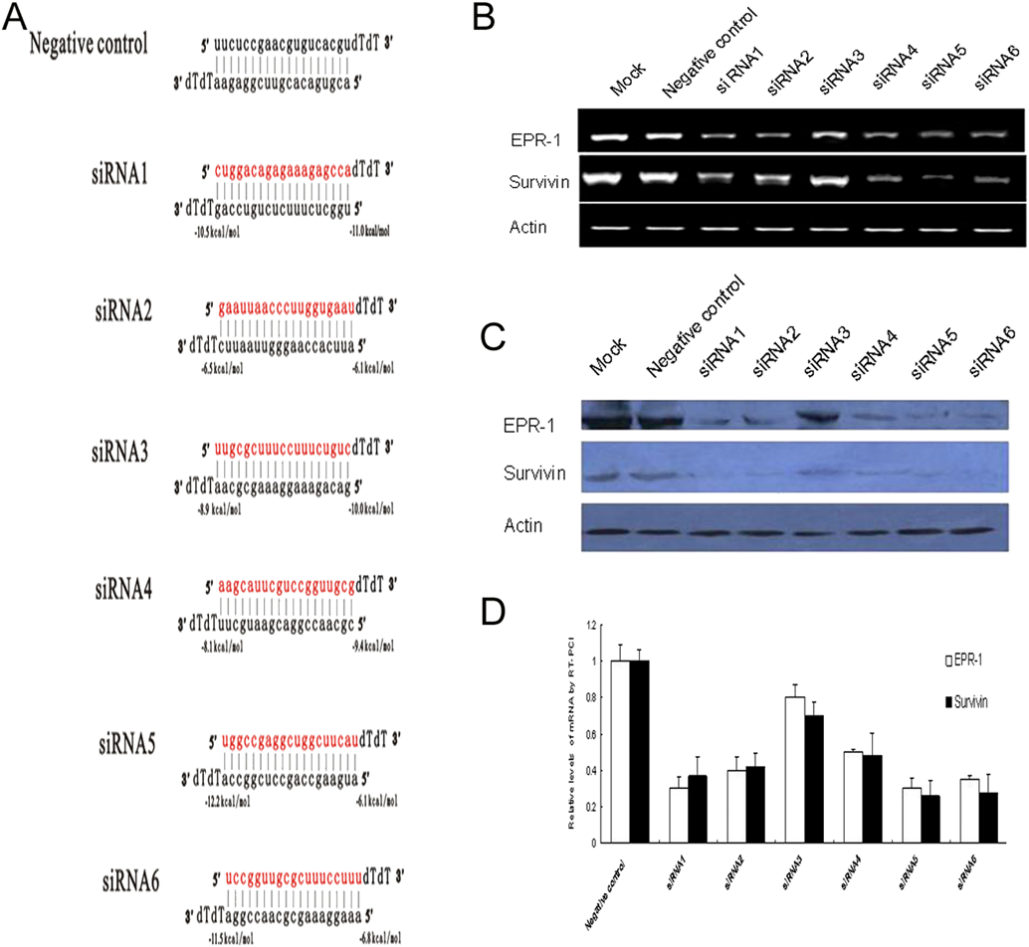
The function of siRNAs targeting the complementary region of EPR-1 and Survivin. (A) Sequences of siRNAs. The sense strands of siRNAs were designed against the mRNA of the Survivin. The predicted melting free energies of the 4 terminal base pairs of each siRNA are indicated. Throughout this paper, antisense siRNA strands have been presented in black and sense siRNAs strands in red. (B, D) Twenty-four hours after HEK293 cells were transfected with siRNA, the degradation of EPR-1 and Survivin was analyzed by RT-PCR and qRT-PCR using β-actin for standardization. (C) Forty-eight hours after HEK293 cells were transfected with siRNA, the expressions of EPR-1 and Survivin were analyzed by Western blotting using β-actin for standardization.

**Figure 3:**
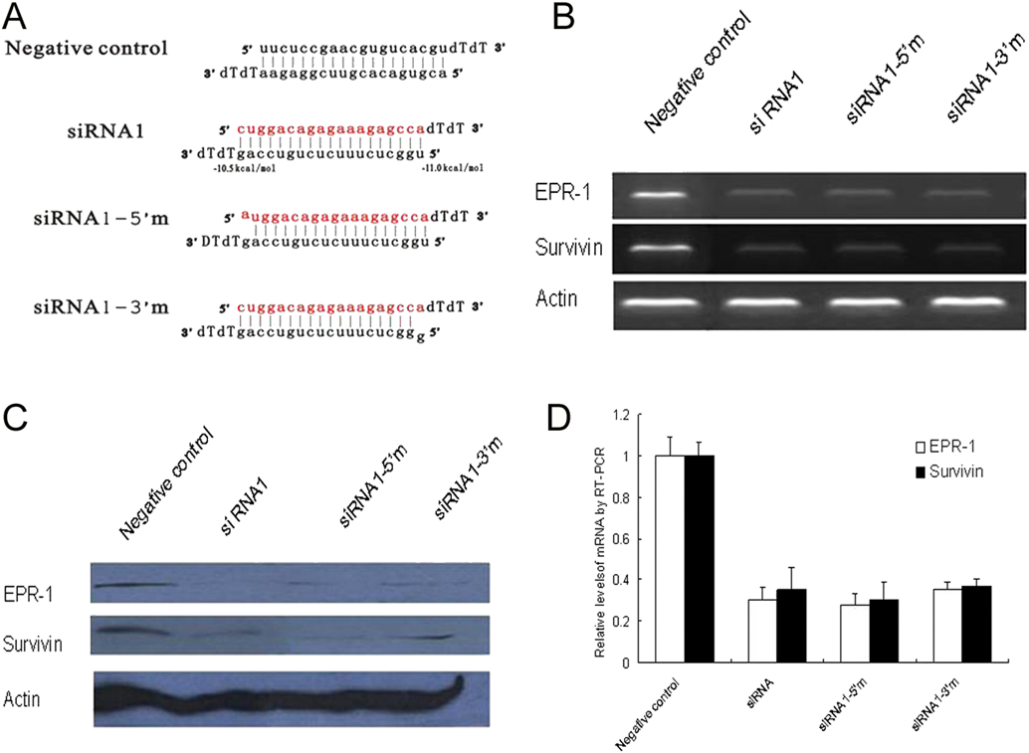
Terminal, single-nucleotide mismatches do not alter the function of siRNA in cultured mammalian cells. (A) The sequences of siRNA and mismatched siRNA. The predicted melting free energies of the 4 terminal base pairs of sense and antisense strands are similar to those of completely matched siRNA1. The first nucleotide of 5′ end of the sense and antisense strands were mismatched individually (siRNA1-5′m and siRNA1-3′m). (B, D) Twenty-four hours after HEK293 cells were transfected with siRNA1 and mismatched siRNA1, the degradation of EPR-1 and Survivin was analyzed by RT-PCR and qRT-PCR using β-actin for standardization. (C) Forty-eight hours after HEK293 cells were transfected with siRNA1 and mismatched siRNA1, the expressions of EPR-1 and Survivin were analyzed by Western blotting using β-actin for standardization.

In order to assess whether only the guide strand of the siRNA duplex is competent in directing RNAi and if the selection of the guide strand always follows the rule of thermodynamic asymmetry in cultured mammalian cells, we measured the cleavage rates of the siRNA strand whose sequence was identical to that of the targeted Survivin mRNA sites (sense strand) and the strand complementary to the corresponding Survivin mRNA sequence (antisense strand). All siRNA duplexes were targeted to the complementary regions of EPR-1 and Survivin. For siRNA with different thermodynamic profiles at either the 5′ or 3′ end of the siRNAs, the degradation of both EPR-1 and Survivin was observed in our experiment (Figure 2). To further verify the influence of thermodynamic profiles of siRNA duplexes on the selection of the guide strand, we synthesized two types of siRNA duplexes containing one nucleotide mismatch at either the 5′ or 3′ end; both siRNAs corresponded to siRNA1 (Figure 3). The results showed that the siRNA duplexes with either a 5′ or 3′ end mismatch did not alter the degradation of EPR-1 or Survivin, indicating that changing the thermodynamic profiles of the siRNA duplex does not influence the selection of the guide strand.

### EPR-1/Survivin siRNA guide strand selection is independent of variation in the siRNA thermodynamic profile and Dicer processing polarity in cultured mammalian cells

**Figure 4:**
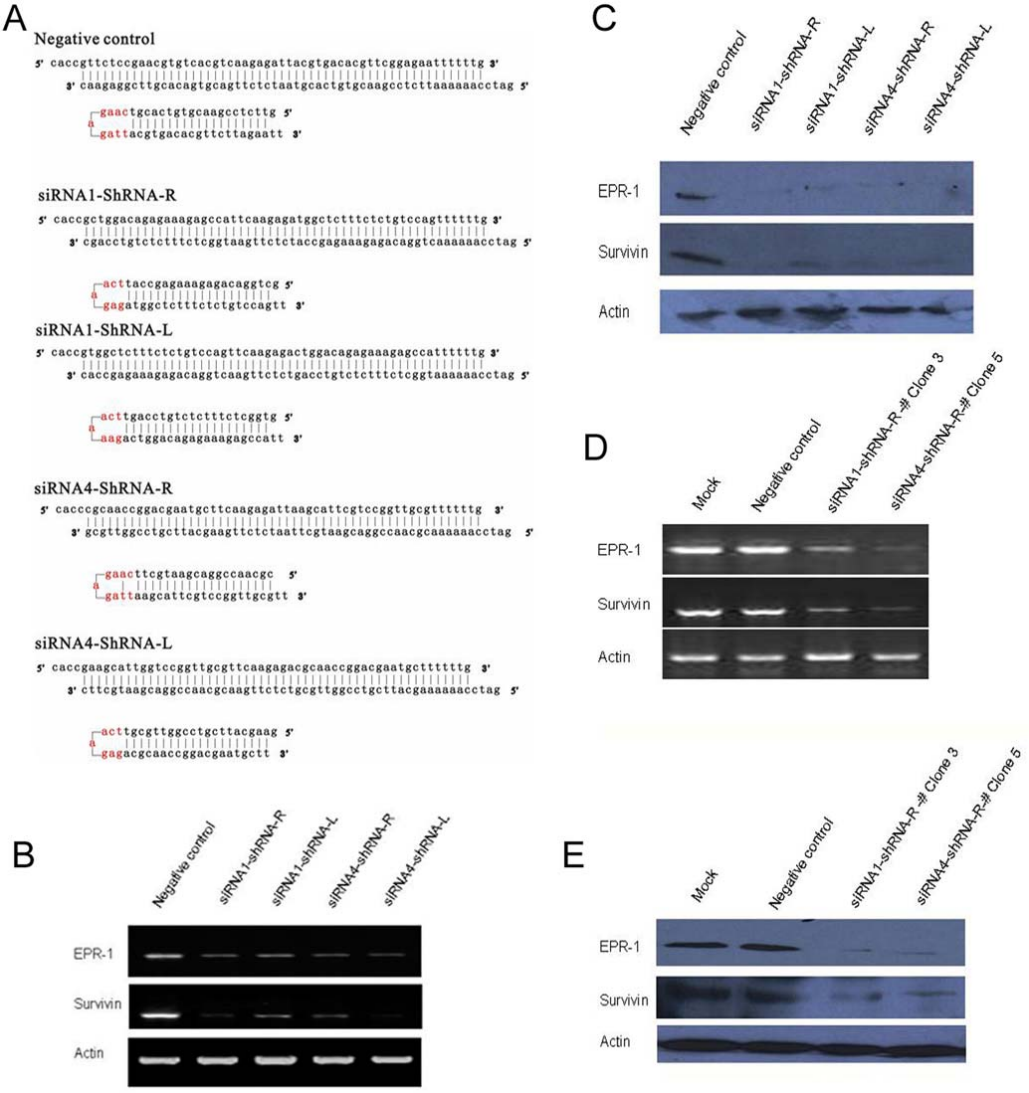
The direction of Dcr processing does not alter the selective property of the guide strand. (A) The sequences of shRNAs. The core 19 nt were obtained from siRNA1 and siRNA4 as mentioned above. The predicted melting free energies of the 4 terminal base pairs of the sense and antisense strands are similar to those of siRNA1. While in siRNA4, the energy difference between the 5′ end of sense and antisense is approximately −1.4 kal/mol. shRNA-R and shRNA-L permit processing specifically from either the 5′ or 3′ end of the incipient siRNA1 or siRNA4. (B) Twenty-four hours after HEK293 cells were transfected with shRNAs, the expressions of EPR-1 and Survivin were analyzed by RT-PCR using β-actin for standardization. (C) Forty-eight hours after HEK293 cells were transfected with shRNA, the expressions of EPR-1 and Survivin were analyzed by Western blotting using β-actin for standardization. (D) The expressions of EPR-1 and Survivin in single cell clones expanded from the siRNA1-ShRNA-R and siRNA4-ShRNA-R were analyzed by RT-PCR using β-actin for standardization. (E) The expressions of EPR-1 and Survivin in single cell clones expanded from the siRNA1-ShRNA-R and siRNA4-ShRNA-R were analyzed by Western blotting using β-actin for standardization.

During dsRNA processing, Dcr releases siRNA from dsRNA ends in a manner dictated by asymmetric enzyme-substrate interactions [3], [16]. To test whether processing polarity can affect strand selection in cultured mammalian cells, we constructed vectors that transcribe short hairpin RNAs (shRNAs); in these shRNAs derived from siRNA1 and siRNA4, the stem loop was located at either flank of the original siRNAs, permitting processing specifically from sites corresponding to either the 5′ or 3′ end of the sense strand of the incipient siRNA (Figure 4). The shRNAs that form loops at the site corresponding to the 2-nt 3′-overhanging end of the original sense or antisense strand were referred to “shRNA-R” or “shRNA-L”, respectively. For the shRNA counterparts of siRNA1, the selection of the siRNA strand with the exposed 3′ end would not be favored based on the thermodynamic asymmetry rule. In the case of siRNA4-based shRNAs, the loop in shRNA-R would force Dcr to initiate binding preferably from the site corresponding to the more weakly base-paired flank of the original siRNA, while in shRNA-L Dcr would bind the site corresponding to more stable flank of the siRNA4. At the same time, we included these two shRNAs with the aim of testing whether dsRNA processing could impart asymmetric strand selection in a processed siRNA that was thermodynamically symmetric or asymmetric.

We transfected the shRNA vectors into HEK293 cells and examined the expression of EPR-1 and Survivin by reverse transcription-polymerase chain reaction (RT-PCR) and Western blotting. In all cases, shRNA-R and shRNA-L exhibited similar efficiency in cleaving their corresponding targets. Both pairs of shRNA-R and shRNA-L showed similar efficiency in guiding the cleavage of their respective sense and antisense targets. Therefore, it is not likely, at least for the EPR-1/Survivin siRNA studied here, that the direction of Dcr processing influences the selection of the guide strand.

To rule out the saturation of the enzymatic machinery involved in RNAi by high concentrations of shRNAs in our system, we obtained stable siRNA-1-R- and siRNA-4-R-expressing cell clones, which showed comparably suppressed expression of both EPR-1 and Survivin. These results suggest that both strands of the EPR-1/Survivin siRNA can cause the degradation of homogeneous gene.

### 2′-fluoro-uridine/2′-fluoro-cytidine modification of the EPR-1/Survivin siRNA strands does not alter their functions in RNAi

**Figure 5:**
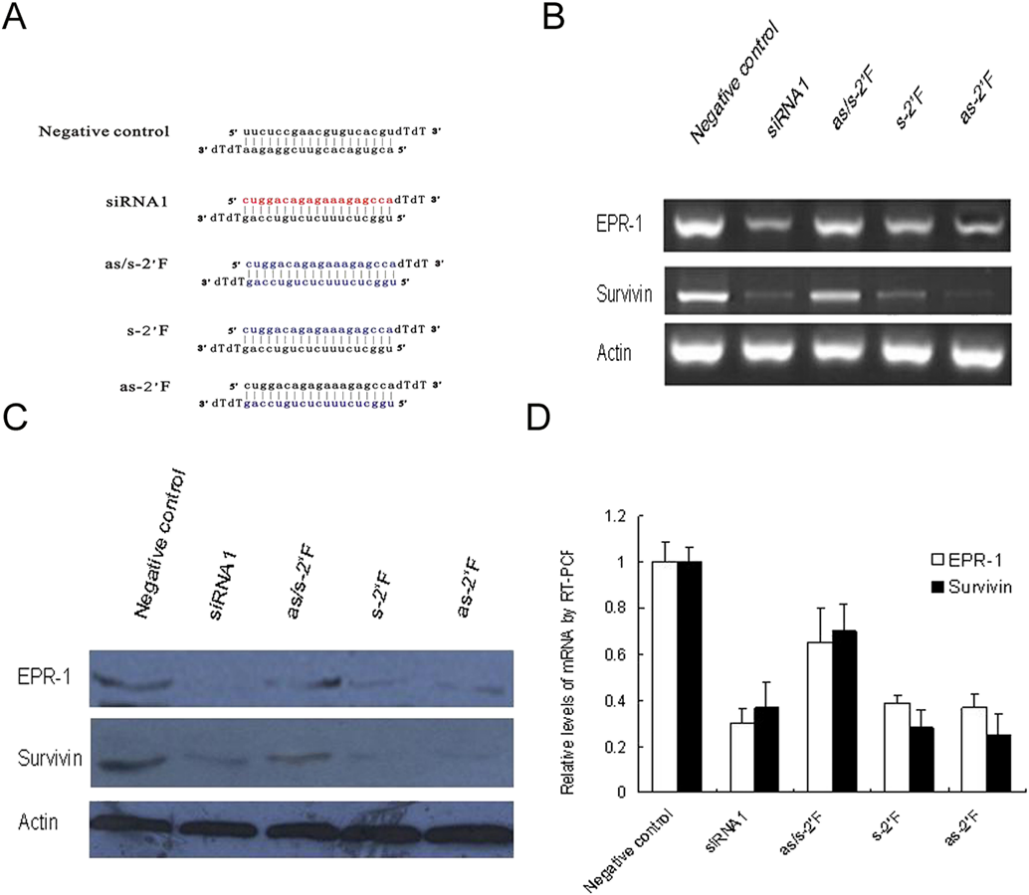
2′ FC, 2′ FU modification of the sense or antisense strand of siRNA does not affect the selective property of the guide strand. (A) The sequences of unmodified and modified siRNAs. as/s-2′ F indicates that both the sense and antisense strands of siRNA1 were modified with 2′ FC, 2′ FU, while in s-2′ F or as-2′ F, only the sense or antisense strands was modified with 2′ FC, 2′ FU, respectively. (B, D) Twenty-four hours after HEK293 cells were transfected with unmodified and modified siRNA1, the expressions of EPR-1 and Survivin were analyzed by RT-PCR and qRT-PCR using β-actin for standardization. (C) Forty-eight hours after HEK293 cells were transfected with unmodified and modified siRNA1, the expressions of EPR-1 and Survivin were analyzed by Western blotting using β-actin for standardization.

2′-fluoro-cytidine (2′ FC) and 2′-fluoro-uridine (2′ FU)-modification of ribose residues in RNA, which greatly increases the stability and prolonged the half-life of RNAs in human plasma as compared to 2′-OH in normal siRNAs, can be used for the site-specific blocking of the activity of ribonuclease including RISC toward RNA targets [2], [8], [17], [18]. In our study, the 2′ FU, 2′ FC modification was introduced in the sense or/and antisense strands of siRNA1 duplexes. For s-2′ F, as-2′ F or as/s-2′ F, the 10^th^ and 11^th^ nucleotides of the sense or antisense strands, or both strands were modified with 2′-fluoro, respectively (Figure 5). According to a reported mechanism in which the cleavage of anti-guide strand is required for release of the siRNA guide strand, the sense strand in as-2′ F and both strands in as/s-2′ F siRNAs would lose their ability to be incorporated into RISC and thus become deficient in triggering RNAi.

We assayed the degradation of EPR-1 and Survivin after transfecting the HEK293 cells with the modified siRNA duplex. Our data showed that the 2′ FU, 2′ FC modification of either the sense or antisense strand of siRNAs did not alter their potential for silencing. When both strands of the siRNA duplex were modified, we observed a remarkably decreased but still significant inhibition of EPR-1 and Survivin expression (Figure 5). Thus, the marked decrease in the RNAi efficiency following the modification of both strands may be possibly attributed to steric hindrance toward the enzymes involved in the unwinding of the siRNA duplex. These results suggest that alternative mechanisms other than the cleavage event occurring within the siRNA duplex are responsible for the elimination of the anti-guide strand from the RISC and the degradation of the mRNA substrate in our study.

### Both strands of the EPR-1/Survvin or caspase siRNA duplex can be incorporated during Ago2-TRBP-dependent RISC formation

**Figure 6:**
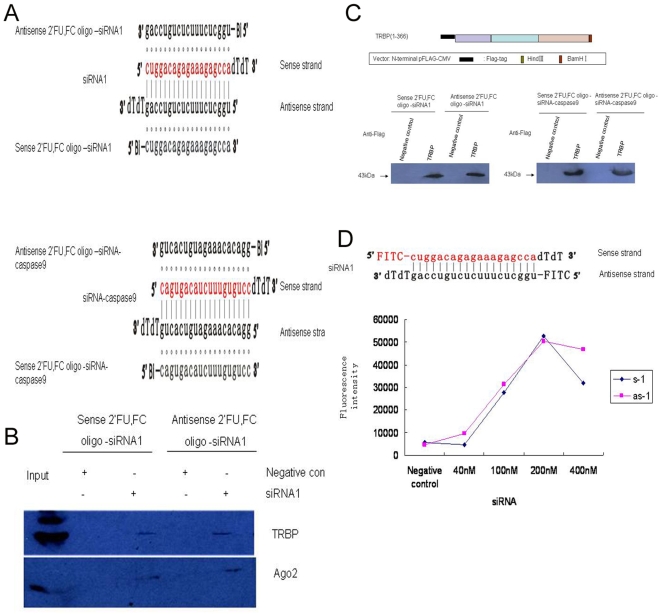
Capture of RISC by both s-2′ FU, 2′ FC oligonucleotides and as-2′ FU, 2′ FC oligonucleotides. (A) Sequences of the s/as-2′ FU, 2′ FC oligonucleotides and siRNAs. (B) Analysis of RISC capture by Western blotting using anti-TRBP (*top*) and anti-Ago2 (*bottom*) antibodies. (C) Schematic representation of TRBP construct with a Flag tag. The numbers indicate amino acid loci. After cotransfecting the HEK293 cells with siRNA1 (targeting both EPR-1 and Survivin) or siRNA-caspase9 (targeting caspase-9) and TRBP, the active RISCs were captured by beads preincubated with 2′ FU, 2′ FC oligonucleotides. Then, the proteins associated with the siRNA-2′ FU, 2′ FC oligonucleotides were recovered and analyzed by Western blotting using an anti-Flag antibody. (D) The sequences of FITC modified siRNA1. After transfecting the HEK293 cells with FITC-siRNA1, the active RISCs were captured by beads preincubated with s/as-2′ FU, 2′ FC oligonucleotides and analyzed by MultisKan.

In an in vitro system, the 2′-*O*-methylated oligonucleotides tethered to streptavidin paramagnetic beads via a 5′ biotin linkage can be used to deplete siRNA-programmed RISC from the reaction. This reaction is based on nucleotide complementarity [19]. In this study, we used 2′ FU, 2′ FC-modified oligonucleotides complementary to the sense and antisense strands of synthetic siRNAs to capture the siRNA-programmed RISC in vivo (Figure 6A). Detection of Ago2 and TAR RNA-binding protein (TRBP) in the captured complex confirmed the capture of active RISC by 2′ FU, 2′ FC-modified oligonucleotides (Figure 6B). In theory, if only one strand acting as the guide strand is incorporated into the active RISC, the 2′ FU, 2′ FC-modified oligonucleotides complementary to the sense strand cannot capture the RISC. Conversely, our results showed that both 2′ FU, 2′ FC oligonucleotides complementary to the sense and antisense strands of siRNA could capture active RISC with Ago2 in vivo (Figure 6B, C and D).

Analysis of the interaction between TRBP, Ago2 and siRNA indicates that TRBP may play a role in mediating the interaction between Dcr and siRNA and the recruitment of Ago2. It is reported that the binding of TRBP to RNAs can enhance RNAi efficiency. To better understand whether TRBP is responsible for the delivery of functional siRNA strand to the protein constituents of RISC, we cotransfected siRNA1 and Flag-tagged truncated TRBPs, which contain three dsRBDs (Figure 6C) into the HEK293 cell lines. Then, to capture the siRNA-programmed RISC in HEK293 cell lines in vivo, we used 2′ FU, 2′ FC-modified oligonucleotides complementary to the sense and antisense strands of siRNA1 that recognizes the EPR-1 and Survivin mRNAs, respectively, and a caspase-9-targeted siRNA containing only one strand that can find its target mRNAs (Figure 6A). Western blot analysis using an antibody against Flag demonstrated that truncated TRBP can be incorporated into the RISC that is loaded with siRNA1, and no significant difference in the TRBP assembly efficiency between sense strand- and antisense strand-precipitated RISCs was observed. Studies using the siRNA against caspase-9 also showed the same tendency (Figure 6C). Taken together, these data imply a comparable opportunity of both strands of the tested siRNA duplexes, regardless of the existence of their target mRNA in the cell, to be incorporated into RISC, a process dependent upon the TRBP protein.

To further access if both strands of siRNA are incorporated into RISC, we modified both 5′ end of siRNA1 sense strand and antisense strand with FITC. After transfecting the modified siRNA into HEK293 cell line, we used 2′ FU, 2′ FC-modified oligonucleotides complementary to the sense and antisense strands of siRNA1 to capture RISC containing sense strand or antisense strand. Again, the results showed that both sense and antisense strands of the Survivin siRNA were incorporated into RISC in HEK293 cells (Figure 6D).

## Discussion

The complementary characteristic of EPR-1 and Survivin provides a natural model for investigating the functions of the siRNA duplex and the formation of RISC in cultured mammalian cells. Survivin expression has been confirmed in some solid tumors, including gastric, colorectal, pancreatic and hepatocellular cancers, sarcoma, and hematologic neoplasias, while the expression of EPR-1 has been detected in a relatively limited number of tumors; the simultaneous expression of EPR-1 and Survivin has been demonstrated by very few studies [9], [20]. By using Western blotting we found that HEK293 was a natural model expressing both EPR-1 and Survivin simultaneously.

Several in vitro observations have suggested that thermodynamic asymmetry governs the strand selection that is necessary for triggering efficient RNAi, in which only the guide strand of the siRNA duplex is incorporated in the RISC [21], [22], [23]. However, the fate of the anti-guide strand and whether small RNA stability is always a factor that determines RISC assembly in mammalian cells is not clear. We observed concurrent degradation of both EPR-1 and Survivin triggered by siRNAs with different thermodynamic profiles targeting to the complementary regions of these two genes. In addition, siRNA duplexes with either a 5′ or 3′ end mismatch did not alter the degradation of EPR-1 and Survivin. A possible explanation is that the two strands of the siRNA duplex are equally loaded onto Ago2 of the active RISC and that the RISC containing strands that are unable to find its target mRNA in cells is degraded. Capture of RISC by either labeled strand of the RNA duplex further confirmed this view. The finding that both strands of the tested siRNA duplexes are capable of forming functional RISC in our study suggests that other undefined features in cultured mammalian cells, in addition to the thermodynamic characters of the siRNA, might play a role in the selection of guide strands.

In mammalian cells, Dcr enzymes are required for RISC assembly as well as dsRNA processing, suggesting that the two phases of RNAi might be functionally coupled in a manner that affects siRNA strand selection. However, our experiments indicate that in HEK293 cells, RISC assembly and siRNA strand selection in EPR-1/Survivin-targeted RNAi are not significantly influenced by the artificial loops in either flank of the shRNA. These data suggest the possibility of siRNA strand selection that does not conform to the thermodynamic asymmetry rule, or alternatively, the uncoupling of shRNA processing resulting in loop removal and subsequent cleavage of the anti-guide strand.

We then explored the influence of the modification of cleavage sites to the RNAi activity. Our results showed that the 2′ FU, 2′ FC modification of either the sense or antisense strand of siRNAs did not alter their potential for gene silencing. Whereas our findings on EPR-1/Survivin siRNA may suggest that the cleavage event is dispensable for the anti-guide strand to leave the RISC and activate the cleavage of the mRNA substrate, or alternative mechanisms are responsible for removing the anti-guide siRNA from RISC, we cannot rule out the incomplete blockade of anti-guide strand cleavage by 2′-fluoro-uridine and cytidine modification.

The association of the Dcr-TRBP complex with Ago2 is suggestive of the physical coupling of the RNAi initiation step with the ultimate RNA targeting and gene silencing. It has also been demonstrated that the complex containing TRBP, Dcr, and Ago2 is required for microRNA (miRNA)/siRNA biogenesis and links the miRNA/siRNA processing to the assembly of an active RISC [24], [25], [26]. Given that Ago2 is unable to bind the siRNA complex on its own, TRBP may play a role in mediating the interaction between Dcr and siRNA and the recruitment of Ago2. It is reported that Dicer and TRBP interact through the carboxy-terminal domain of TRBP in a yeast two-hybrid assay [26]. In this study, in the attempt to dissect whether TRBP is responsible for the incorporation of guide strand to the protein constituents of RISC, we found that TRBP could be incorporated into the RISC captured by both strands of siRNA duplex in the presence or absence of their target RNAs in the cells, suggesting that TRBP does not influence the selection of guide strand. Taken together, the present studies on EPR-1/Survivin and caspase-9 siRNAs imply the coordinated incorporation of both siRNA strands into the RISC during RNAi, regardless of the presence or absence of their target RNAs in the cells.

### A hint for the RNAi mechanism

Since the discovery of the remarkable RNAi phenomenon, many scientists have focused on understanding the underlying mechanism better and utilizing RNAi as a tool for genetic and therapeutic studies. It is now clear that RNAi occurs in evolutionarily diverse organisms. Both long dsRNAs and shRNAs are converted by the RNase III endonuclease Dcr to ∼21 nt siRNA containing 2-nt 3′ overhangs. By incorporated into a protein complex, the siRNA duplex becomes a component of the RISC and mediates the cleavage of target RNAs [24], [27]. However, the manner in which the siRNA duplex is loaded onto the RISC and the fate of the anti-guide strand that is unable to find its target mRNA remain elusive.

Here, using siRNAs targeted to caspase-9 and the complementary region of EPR-1 and Survivin, our study indicates that both strands of the siRNA duplex can be loaded onto Ago2 of the active RISC in HEK293 cells, and that the unwinding of the siRNA is not impaired by a fluoro modification on the ribose of the anti-guide strand, which is believed to block cleavage by RNases. In addition, we found that an siRNA duplex that did not conform to the rule of thermodynamic asymmetry could also induce the silencing of EPR-1 or Survivin, indicating that in this case machineries beyond the siRNA sequences determine the initiation and effectiveness of RNAi. This was also demonstrated by additional evidence in our further studies using modified siRNAs. We assume that whereas the guide strand selection on most RNA interference reactions may follow the thermodynamic asymmetry rule in mammalian cells, this does not exclude the possibility for a certain class of siRNAs that two types of active RISC, each containing one of the complementary strands, are formed in response to one siRNA duplex. Subsequently, RISCs that can find their target mRNAs directs the degradation of the target mRNA, while those which cannot find their target mRNA may be degraded by a yet unknown pathway. Clark PR et al [28] found that an siRNA molecule selected to target ICAM-1 through its antisense strand exhibited broad anti-TNF activities. They also showed that this “off-target” effect was mediated by siRNA knockdown of TNFR 1 via its sense strand. Our results indicate that the incorporation of sense strand into active RISC leads to the degradation of TNFR in their study. Ro S et al [29] found that the sense and antisense strands could co-accumulate as miRNA pairs in some tissues while being subjected to strand selection in other tissues. In addition, the loading of miRNA did not always follow the rule of thermodynamic asymmetry. Just at the moment of finishing our paper, the results from another laboratory using cell-based RNAi assays and large-scale RNAi data analyses provided additional support to our conclusion, demonstrating that there is no definite correlation between siRNA stability and gene silencing in mammalian cells [30]. Considering that it is generally accepted that siRISC and miRISC are functionally interchangeable, the common characteristic of siRNA and miRNA loading indicates that the role of single strand features might be overestimated in determining its probability of incorporation into an active RISC.

## Materials and Methods

### Design and preparation of siRNAs

siRNAs complementary to EPR-1 and Survivin were designed to target 19 nt sequences beginning from the following positions: siRNA1, E752/S431; siRNA2, E779/S404; siRNA3, E813/S370; siRNA4, E827/S356; siRNA5, E949/S234; and siRNA6, E818/S365. The predicted melting free energies of the 4 terminal base pairs of both 5′ and 3′ termini of the siRNA duplex were calculated based on the Sfold server algorithm (http://sfold.wadsworth.org/sirna.pl). Synthetic siRNAs, including those with the 2′-fluoro (2′ F)-modification, were manufactured by Genepharma Inc.

### Construction of the siRNA expression vector pGPU6/GFP/Neo

The target sequences of siRNA1 and siRNA4 in the complementary region of EPR-1 and Survivin were selected for further shRNA targeting. Synthesized oligodeoxyribonucleotides encoding the corresponding siRNAs were designed as follows: 19 nt target sequence as sense strand was followed by a 9 nt spacer and complementary antisense strand, with six repeated Ts as transcription termination signal. For negative control, double-stranded oligonucleotide, which contained a 19 nt sequence with the same nucleotide composition as siRNA1 and siRNA4 but lacks significant sequence homology to the human genome, was introduced into linearized vector pSuNeo. All paired oligodeoxyribonucleotides were annealed and ligated into a BamHI- and BbsI -digested pSuNeo vectors. Positive clones were confirmed by sequencing.

### Cell culture and transfection

The HEK293 cell line was purchased from ATCC and was cultured in Dulbecco's modified Eagle's medium (DMEM containing high glucose level; GIBCO BRL) supplemented with 10% fetal bovine serum (FBS, Invitrogen). Then, 6-well plates (Becton Dickinson) were seeded at an initial density of 0.8×10^5^ cells/well on the day prior to transfection and incubated at 37°C and in 5% CO_2_. Synthetic siRNAs and the siRNA expression vector pGPU6/GFP/Neo were delivered to cells (typically at 40%–60% confluence) using a Lipofectin reagent (Invitrogen) according to the manufacturer's instructions. To obtain stably transfected clones, cells were selected with 400 ug/mL G418 (Life Technologies) for 3 to 4 weeks and single clones were isolated.

### RNA isolation, semi-quantitative RT-PCR, and quantitative RT-PCR

Total RNA was extracted from HEK293 cells by using TRIzol reagent according to the manufacturer's instructions. Complementary DNA (cDNA) was generated from total RNA by reverse transcription using Moloney murine leukemia virus (M-MLV). PCR amplification of the cDNA was performed in a reaction mixture with a final volume of 30 µL containing 2 µL of 4×dNTPs, one unit of *Taq* DNA polymerase, and 10 mmol/L of each paired primer specific to EPR-1 and Survivin, respectively.

For the semi-quantitative RT-PCR, the primers used for EPR-1 amplification were as follows: forward primer, 5′-ATGACCTCCAGAGGTTTCCA-3′ and reverse primer, 5′-TGCCATCGAGCAGCTGGCCT-3′. Those used for Survivin were as follows: forward primer, 5′-TTTCTCAAGGACCACCGCATCT-3′ and reverse primer, 5′-GCCTAAGAGGGCGTGCGCT-3′. The amplification protocol followed was 25 cycles at 95°C for 30 s, 58°C for 30 s, and 72°C for 1 min, and finally at 72°C for 10 min. The PCR products were separated by electrophoresis on 15 g/L agarose gels and visualized by ethidium bromide staining. Amplified human β-actin served as the control for sample loading and integrity.

For quantitative RT-PCR (qRT-PCR), PCR was performed with the Access qRT-PCR Kit (TaKaRa) according to the manufacturer's instructions. The primers used for qRT-PCR of EPR-1 were forward primer, 5′-TTCTCAAGGACCACCGCATC-3′ and reverse primer, 5′-GCCAAGTCTGGCTCGTTCTC-3′. Those used for Survivin were forward primer, 5′-AGGTCATCTCGGCTGTTCCTG-3′ and reverse primer, 5′-TCATCCTCACTGCGGCTGTC-3′. All primers were purchased from TaKaRa.

### Western blotting

After protein quantification, the cell extract was resolved by 10% sodium dodecyl sulfate-polyacrylamide gel electrophoresis (SDS-PAGE) and electroblotted onto nitrocellulose membranes (Bio-Rad). The membranes with the transferred proteins were incubated with rabbit anti-human EPR-1 antibody (1∶500, Merck) as the primary antibody, followed by incubation with horseradish peroxidase-conjugated goat anti-rabbit immunoglobulin (IgG) (1∶2,000) as the secondary antibody. Chemiluminescence reaction was carried out with an ECL kit (Pierce) for 1 min, followed by exposure to a Kodak X-Omat radiographic film. Similar procedures were carried out to detect the Survivin protein using an antibody from Abcam (1∶500) and a -actin antibody (1∶500; Santa Cruz) was used for actin blotting.

### Capture of RISC

Immobilized, biotinylated 2′ FU, 2′ FC oligonucleotides (100 pmol) were incubated for 1 h on ice in a lysis buffer containing 2 mM dithiothreitol (DTT) with 50 µL of Dynabeads M280 (Invitrogen). For RISC capture assays, siRNAs were transfected into HEK293 cells using Lipofectamine 2000 (Invitrogen). For the 6-well plate assay, the cell pellet was resuspended in 150 µL of lysis buffer (150 mM NaCl, 0.5% Nonidet P (NP)-40, 2 mM MgCl_2_, 2 mM CaCl_2_, and 20 mM Tris (pH 7.5)) containing Complete Mini Protease Inhibitor Tablet (Roche) and 1 mM DTT. Then, the immobilized 2′ FU/2′ FC oligonucleotides were incubated with the cell lysate for 1 h at 25°C. After incubation, beads were collected using a magnetic stand (Invitrogen). The beads were then washed 3 times with ice-cold lysis buffer containing 0.1% (w/v) NP-40 and 2 mM DTT, followed by washing with a lysis buffer without NP-40.

To recover the proteins associated with the siRNA-2′ FU, 2′ FC oligonucleotides, the beads were boiled for 10 min in 20 µL of SDS loading buffer (10 mM Tris-HCl [pH 6.8], 2% [w/v] SDS, 100 mM DTT, and 10% [v/v] glycerol). Proteins were resolved by SDS-PAGE on an 8% gel and transferred to nitrocellulose membranes (Bio-Rad). To detect the RISC constituent proteins, the membrane was incubated overnight at 4°C with rabbit polyclonal anti-TRBP (1∶250; Imgenex), rabbit polyclonal anti-Ago2 (1∶250; Abcam), or rabbit anti-Flag (1∶1000; Sigma) antibodies, followed by incubation with the appropriate secondary antibodies and band visualization.
